# Retraction note: Up-regulation of miR-130b expression level and down-regulation of miR-218 serve as potential biomarker in the early detection of human osteosarcoma

**DOI:** 10.1186/s13000-016-0565-4

**Published:** 2016-11-02

**Authors:** Afshin Taheriazam, Amir Jouya Talaei, Mohammad Jamshidi, Mohammadreza Shakeri, Samaneh Khoshbakht, Emad Yahaghi, Marjan Shokrani

**Affiliations:** 1 Department of Orthopedics Surgery, Tehran Medical Sciences Branch, Islamic Azad University, Tehran, Iran; 2 Department of Genetics, Faculty of Life Sciences, Azad University of Tehran Medical Sciences Branch, Tehran, Iran; 3 Cellular and Molecular Research Center, Zahedan University of Medical Sciences, Zahedan, Iran; 4 Department of Clinical Biochemistry, School of Medicine, Zahedan University of Medical Sciences, Zahedan, Iran; 5 Department of Orthopaedic and Trauma Surgery, Birjand University of Medical Sciences, Birjand, Iran; 6 Department of Statistics, Faculty of Sciences, Islamic Azad University, Mashhad Branch, Mashhad, Iran; 7 Department of Molecular Biology, Baqiyatallah University of Medical Sciences, Tehran, Iran; 8 Graduate, Faculty of Medicine, Tehran University of Medical Sciences, Tehran, Iran

## Retraction

The Editor-in-Chief and Publisher have retracted this article [1] because the scientific integrity of the content cannot be guaranteed. An investigation by the Publisher found it to be one of a group of articles we have identified as showing evidence suggestive of attempts to subvert the peer review and publication system to inappropriately obtain or allocate authorship. This article showed evidence of plagiarism (most notably from the article cited [2]) and authorship manipulation.
